# Prevalence of Angiodysplasia Detected in Upper Gastrointestinal Endoscopic Examinations

**DOI:** 10.7759/cureus.14353

**Published:** 2021-04-07

**Authors:** Takumi Notsu, Kyoichi Adachi, Tomoko Mishiro, Kanako Kishi, Norihisa Ishimura, Shunji Ishihara

**Affiliations:** 1 Health Center, Shimane Environment and Health Public Corporation, Matsue, JPN; 2 Second Department of Internal Medicine, Shimane University Faculty of Medicine, Izumo, JPN; 3 Gastroenterology, Shimane University Hospital, Izumo, JPN

**Keywords:** angiodysplasia, angioectasia, etiology, endoscopy, hereditary hemorrhagic telangiectasia, osler-weber-rendu syndrome

## Abstract

Background

This study was performed to examine the prevalence of asymptomatic angiodysplasia detected in upper gastrointestinal endoscopic examinations and of hereditary hemorrhagic telangiectasia (HHT) suspected cases.

Methodology

The study participants were 5,034 individuals (3,206 males, 1,828 females; mean age 53.5 ± 9.8 years) who underwent an upper gastrointestinal endoscopic examination as part of a medical check-up. The presence of angiodysplasia was examined endoscopically from the pharynx to duodenal second portion. HHT suspected cases were diagnosed based on the presence of both upper gastrointestinal angiodysplasia and recurrent nasal bleeding episodes occurring in the subject as well as a first-degree relative.

Results

Angiodysplasia was endoscopically detected in 494 (9.8%) of the 5,061 subjects. Those with angiodysplasia lesions in the pharynx, larynx, esophagus, stomach, and duodenum numbered 44, 4, 155, 322, and 12, respectively. None had symptoms of upper gastrointestinal bleeding or severe anemia. Subjects with angiodysplasia showed significant male predominance and were significantly older than those without. A total of 11 (0.2%) were diagnosed as HHT suspected cases by the presence of upper gastrointestinal angiodysplasia and recurrent epistaxis episodes from childhood in the subject as well as a first-degree relative.

Conclusions

Asymptomatic angiodysplasia was detected in 9.8% of the subjects who underwent screening upper gastrointestinal endoscopic examinations.

## Introduction

Gastrointestinal angiodysplasia, also termed angioectasia, is a vascular malformation composed of dilated and tortuous arterial or venous capillaries, usually smaller than 5 mm in diameter, and located in the mucosal and submucosal layers of the gastrointestinal tract [1,2]. It has been reported that gastrointestinal angiodysplasia is responsible for 4-7% cases of nonvariceal upper gastrointestinal bleeding [3-5]. However, nearly all cases of gastrointestinal angiodysplasia are asymptomatic, and it is generally found incidentally in subjects who undergo endoscopic examinations. While Foutch et al. noted that the prevalence of colonic angiodysplasia in healthy asymptomatic individuals was 0.83% [6], its prevalence in the upper gastrointestinal tract in these individuals has not been determined, though recent advances in endoscopic equipment may facilitate better detection of gastrointestinal angiodysplasia.

Hereditary hemorrhagic telangiectasia (HHT), also termed Osler-Weber-Rendu syndrome, is an inherited autosomal dominant vascular disease with varying clinical manifestations, such as epistaxis, gastrointestinal bleeding, and iron deficiency anemia [7]. HHT is considered to be a clinically important disease as associated arteriovenous malformations (AVMs) in cerebral, pulmonary, and hepatic circulation also occur. A diagnosis of HHT is based on the presence of at least three of the following clinical diagnostic findings, known as the “Curacao criteria”: (1) spontaneous recurrent epistaxis; (2) mucocutaneous telangiectasia in characteristic sites, such as the nose, lips, oral cavity, and fingertips; (3) visceral AVM in the lungs, liver, gastrointestinal tract, brain, and spiral cord; and (4) family history of a first-degree relative in whom HHT has been diagnosed based on these criteria [8]. Therefore, HHT might be present in subjects with angiodysplasia detected by an upper gastrointestinal endoscopic screening examination. However, the prevalence of HHT in patients with upper gastrointestinal angiodysplasia has not been investigated.

In this study, we analyzed the prevalence of asymptomatic angiodysplasia in subjects who underwent an upper gastrointestinal endoscopic examination as part of an annual medical check-up as those who undergo such check-ups are considered to be similar to the general population. In addition, the prevalence of HHT suspected cases was also examined among subjects with angiodysplasia detected by the examination. It has been speculated that there may be many in the general population with HHT who have not been diagnosed [7,8]. For the present analysis, HHT suspected cases were determined when episodes of recurrent epistaxis were observed from childhood in not only the subjects with upper gastrointestinal angiodysplasia but also in a first-degree relative.

## Materials and methods

The study subjects were individuals who visited the Health Center of Shimane Environment and Health Public Corporation for a detailed medical check-up between April 2019 and March 2020, the majority of whom were socially active and productive, and were considered to be socioeconomically middle class. During the study period, 5,061 underwent an upper gastrointestinal endoscopic examination. Of those, 27 with post-gastrectomy status were excluded, and the present study cohort was composed of 5,034 subjects (males 3,206, females 1,828; mean age 53.5 ± 9.8 years).

**Figure 1:**
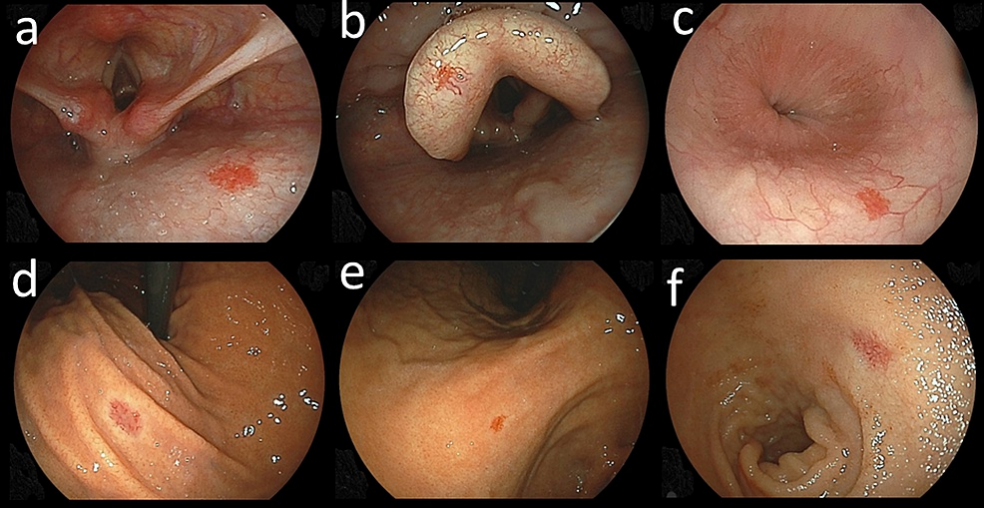
Endoscopic images of angiodysplasia (a: pharynx; b: larynx; c: esophagus; d and e: stomach; f: duodenum).

All upper gastrointestinal endoscopic examinations were performed by experienced licensed endoscopists using an EG-L580NW endoscope (Fujifilm, Tokyo, Japan). At our institution, an upper gastrointestinal endoscopic examination is performed with the subject in an unsedated condition with the endoscope typically inserted in a transnasal manner. Pharyngeal and laryngeal endoscopic observations are more easily performed by transnasal endoscopy than peroral examinations, as the number of gagging episodes during a transnasal endoscopic procedure is known to be significantly less [9]. The presence of angiodysplasia was examined from the pharynx to duodenal second portion during the upper gastrointestinal endoscopic examination in all investigated cases (Figure 1).

All angiodysplasia lesions were recorded on a medical sheet related to the examination. Each endoscopic image from the subject was simultaneously reviewed by three expert endoscopists to confirm the presence of angiodysplasia.

In cases with angiodysplasia, details of any previous episodes of recurrent nasal bleeding noted by the subject as well as in their first-degree relatives were noted in an interview. HHT suspected cases were diagnosed based on the presence of both upper gastrointestinal angiodysplasia and recurrent nasal bleeding episodes in both the subject and a first-degree relative [7,8].

Statistical analyses were performed using a chi-square test, Fisher’s exact probability test, and Mann-Whitney U test. All calculations were done with the StatView 5.0 software program for Macintosh (Abacus Concepts Inc., Berkeley, CA, USA), with a p level of <0.05 considered to indicate statistical significance.

This study was performed in accordance with the Declaration of Helsinki, and the protocol was approved by the ethics committee of the Shimane Environment and Health Public Corporation (IRB No. 2018-1). Written informed consent indicating that the obtained clinical data would be used for a clinical study without release of individual information was received from all subjects before performing the medical check-up.

## Results

Angiodysplasia was endoscopically determined in 494 (9.8%) of the 5,034 study subjects. None had symptoms of upper gastrointestinal bleeding or severe anemia. The numbers of subjects with angiodysplasia in the pharynx, larynx, esophagus, stomach, and/or duodenum were 44, 4, 155, 322, and 12, respectively. A total of 765 angiodysplasia lesions were detected in the 494 cases and the numbers of those in the pharynx, larynx, esophagus, stomach, and duodenum were 49, 4, 184, 514, and 14, respectively (Table 1).

**Table 1:** Location of upper gastrointestinal angiodysplasia. Values are expressed as number of subjects or angiodysplasia lesions. Percentages in parentheses indicate ratio of all subjects or lesions.

Location	Subjects (n = 5,034)	Lesions (n = 765)
Pharynx	44 (0.9%)	49 (6.4%)
Larynx	4 (0.1%)	4 (0.1%)
Esophagus	155 (3.1%)	184 (24.1%)
Stomach	322 (6.4%)	514 (67.2%)
Duodenum	12 (0.2%)	14 (1.8%)

Multiple angiodysplasia lesions were observed in 151 (3.0%) subjects, while the presence of angiodysplasia in multiple organs was detected in 42 (0.8%). Characteristics of subjects with and without upper gastrointestinal angiodysplasia are presented in Table 2.

**Table 2:** Characteristics of subjects with and without upper gastrointestinal angiodysplasia. Values are expressed as the mean ± standard deviation or number of subjects. Percentages in parentheses indicate ratio of the cases with and without upper gastrointestinal angiodysplasia ^#1^Habitual drinking: alcohol drinking at three times and over per week. ^#2^Cardiovascular and cerebrovascular: current and previous histories of hypertension, angina, myocardial infarction, cerebral infarction, cerebral hemorrhage, and carotid artery stenosis. ^#3^Valvular diseases of the heart: current and previous histories of valvular diseases of the heart. ^#4^Chronic kidney diseases: current history of chronic kidney diseases. ^#5^Recurrent epistaxis: recurrent epistaxis episodes from childhood. ^#6^Family history: recurrent epistaxis episodes from childhood in a first-degree relative

	With angiodysplasia (n = 494)	Without angiodysplasia (n = 4,540)	P-Value
Gender (male/female)	349/145	2,857/1,683	<0.001
Age (years)	54.3 ± 9.1	53.4 ± 9.8	0.020
Age range (years)			0.009
≤39 (n = 360)	17 (3.4%)	343 (7.6%)	
40-49 (n = 1,435)	142 (28.7%)	1,293 (28.5%)	
50-59 (n = 1,848)	188 (38.1%)	1,654 (36.4%)	
≥60 (n = 1,418)	147 (29.8%)	1,250 (27.5%)	
Habitual drinking^#1^	237 (48.0%)	1,940 (42.7%)	0.026
Habitual smoking	160 (32.4%)	772 (17.0%)	<0.001
Cardiovascular and cerebrovascular diseases^#2^	95 (19.2%)	898 (19.8%)	0.771
Valvular diseases of the heart^#3^	0	10 (0.2%)	0.612
Chronic kidney diseases^#4^	2 (0.4%)	33 (0.7%)	0.574
Recurrent epistaxis^#5^	21 (4.3%)		
Family history^#6^	36 (7.3%)		
Recurrent epistaxis and family history	11 (2.2%)		

Angiodysplasia cases showed significant male predominance and the cases with angiodysplasia were older than those without. The prevalence of angiodysplasia in ≤39, 40-49, 50-59,v and ≥60-year age group was 4.7, 9.9, 10.2, and 10.4%, respectively (p = 0.009). Habitual drinking and smoking were frequently observed in cases with upper gastrointestinal angiodysplasia. There was not significant difference between the cases with and without upper gastrointestinal angiodysplasia in the current and previous histories of cardio-cerebrovascular diseases, valvular diseases of the heart, and chronic kidney diseases.

Of the 494 subjects with angiodysplasia, a recurrent epistaxis episode that occurred in childhood was noted in 21 (4.3%) and a recurrent epistaxis episode in a first-degree relative was reported in 36 (7.3%) cases. Only 11 cases included a subject with recurrent epistaxis from childhood in both the subject and a first-degree relative. Therefore, 11 (0.2%) of the 5,034 study subjects were considered to be HHT suspected cases. Those included seven males (0.22% of male subjects) and four females (0.22% of female subjects), thus gender was not a significant factor for prevalence of HHT suspected status. Four of the 11 HHT suspected cases had multiple angiodysplasia lesions, while none had angiodysplasia in multiple organs. Because the study subjects were individuals who underwent medical check-ups, we recommended that any considered to be HHT suspected visit a specialized medical center to undergo detailed examination for detection of AVMs in several organs. Although none was diagnosed as HHT following detailed examinations, periodic medical examinations were recommended to confirm the presence of the disease in some of those cases.

## Discussion

Gastrointestinal angiodysplasia is an important vascular lesion as associated bleeding is a significant factor related to morbidity [1-5,10-14]. These lesions are considered to be likely responsible for approximately 3-40% of colonic bleeding cases and 50% of the cases with bleeding in the small intestine [10-14]. In addition, 4-7% of cases of nonvariceal upper gastrointestinal bleeding are thought to be caused by angiodysplasia [3-5]. However, nearly all patients with angiodysplasia are asymptomatic and the prevalence of the disease has not been thoroughly investigated in the general population. The present study was conducted to examine the prevalence of angiodysplasia in individuals who underwent an upper gastrointestinal endoscopic examination as part of an annual medical check-up. The results showed that asymptomatic angiodysplasia was detected in 495 (9.8%) of the 5,061 subjects.

Although the etiology of angiodysplasia is not clearly understood, it may be related to aortic stenosis, while mucosal hypoperfusion associated with cardiac disease is reported to cause angiodysplasia [15,16]. Therefore, hypoperfusion of micro-vessels caused by age-related degeneration of small blood vessels is considered to be an important risk factor for angiodysplasia development [17]. The present results demonstrated an age-related increase in the prevalence of upper gastrointestinal angiodysplasia, although the results of this study could not demonstrate positive correlation between the presence of upper gastrointestinal angiodysplasia and cardiovascular, cerebrovascular, and cardiac valvular diseases. In addition, male predominance for the prevalence of angiodysplasia was observed in this study. Gender-related differences in hormonal balance might be related to such male predominance as estrogen has been repeatedly demonstrated to reduce the onset of several different cardiovascular diseases including arteriosclerosis [18]. Habitual drinking and smoking might affect the male predominance of angiodysplasia prevalence. Further study is needed to clarify the influence of gender on the development of angiodysplasia.

To the best of our knowledge, the prevalence of angiodysplasia among different upper gastrointestinal organs has not been reported. In this study, all instances of endoscopically detected angiodysplasias were noted during examinations conducted over a one-year period. Angiodysplasia lesions were observed in the esophagus in 3.1% and the stomach in 6.4% of the subjects. These values for prevalence of esophageal and gastric angiodysplasia in healthy subjects are markedly higher than those for colonic angiodysplasia reported by Foutch et al. (0.83%) [6]. Nevertheless, additional further studies are needed to compare the prevalence of angiodysplasia between upper and lower gastrointestinal organs in the same subject. On the other hand, angiodysplasia lesions were observed in the pharynx, larynx, and duodenum in 0.8, 0.1, and 0.2%, respectively, of our subjects. Endoscopic observation in those areas is considered to be limited and angiodysplasia can be difficult to detect, though we carefully performed the endoscopy procedures to specifically examine for the presence of angiodysplasia in this study. Therefore, the prevalence of angiodysplasia in the pharynx, larynx, and duodenum is thought to be higher than that demonstrated in the present subjects.

In patients with HHT, the prevalence of gastric angiodysplasia has been reported to range 29-75% [19-22], while that of esophageal angiodysplasia was found to be 26% [23]; thus, upper gastrointestinal angiodysplasia is commonly observed in association with HHT. In the general population, the prevalence of HHT is estimated to be between 0.015 and 0.02% [24]. However, the disease is underreported due to variable penetrance and the appearance of only minor symptoms until later age in many patients, resulting in the number of established diagnoses significantly lower than the estimated prevalence [7].

Of the 5,061 subjects, 11 were diagnosed as HHT suspected cases for a prevalence of 0.2%. In addition, further detailed medical examinations were recommended to clarify the presence of HHT disease in some of those cases. Therefore, the presence of angiodysplasia detected by an upper gastrointestinal endoscopic examination is considered to be an important finding for screening for HHT. A future large-scale, long-term study is recommended to clarify the significance of endoscopically detected upper gastrointestinal angiodysplasia for diagnosis of HHT.

This study has several limitations. The subjects were socially active and productive individuals who visited a medical center for a medical check-up. Therefore, the numbers of younger and older people were relatively small, though they are considered to be similar to the general population. Some angiodysplasia lesions may have been missed due to difficulties with endoscopic observation. In addition, the investigation was performed in a cross-sectional manner; thus, we were unable to determine changes in the number and size of the lesions detected. Further investigation is necessary to reveal the natural history of upper gastrointestinal angiodysplasia. A diagnosis of HHT is usually performed by four clinical diagnostic findings of the “Curacao criteria” [8]. In this study, we diagnosed HHT suspected cases based on the presence of upper gastrointestinal angiodysplasia and recurrent epistaxis episodes from childhood in the subject and a first-degree relative. We did not investigate the presence of mucocutaneous telangiectasias and visceral AVMs, although all HHT suspected cases were recommended to visit a specialized medical center to undergo detailed examination for detection of AVMs in several organs. In addition, we were not able to examine several genetic mutations to confirm the presence of HHT as our institution only conducts medical check-ups. Therefore, further large-scale studies are needed to clarify the prevalence of HHT in cases with upper gastrointestinal angiodysplasias detected by endoscopic examination.

## Conclusions

In conclusion, asymptomatic angiodysplasia was detected during an upper gastrointestinal endoscopic examination in 494 (9.8%) of 5,034 subjects, including 155 (3.1%) with lesions shown in the esophagus and 322 (6.4%) with those in the stomach. Cases of angiodysplasia showed significant male predominance and older age. A total of 11 (0.2%) subjects were considered to be HHT suspected cases based on the presence of angiodysplasia, as well as recurrent epistaxis episodes from childhood in the subject and a first-degree relative. The presence of angiodysplasia detected by upper gastrointestinal endoscopy is considered to be an important finding for screening for HHT.
